# Maguari Virus Associated with Human Disease

**DOI:** 10.3201/eid2308.161254

**Published:** 2017-08

**Authors:** Allison Groseth, Veronica Vine, Carla Weisend, Carolina Guevara, Douglas Watts, Brandy Russell, Robert B. Tesh, Hideki Ebihara

**Affiliations:** Friedrich-Loeffler-Institut, Greifswald–Insel Riems, Germany (A. Groseth);; National Institutes of Health, Hamilton Montana, USA (A. Groseth, V. Vine, C. Weisend, H. Ebihara)**;**; US Naval Medical Research Unit 6, Lima, Peru (C. Guevara, D. Watts);; Centers for Disease Control and Prevention, Ft. Collins, Colorado, USA (B. Russell)**;**; University of Texas Medical Branch, Galveston, Texas, USA (R.B. Tesh)**;**; Mayo Clinic, Rochester, Minnesota, USA (H. Ebihara)

**Keywords:** Maguari virus, Cache Valley virus, Tlacotalpan virus, Playas virus, Fort Sherman virus, bunyavirus, orthobunyavirus, phylogeny, human infection, disease, viruses, vector-borne infections, arboviruses, mosquitoes, United States, Mexico, Brazil, Central America, South America, Peru, Panama, Argentina, Ecuador, Paraguay, Colombia, French Guiana

## Abstract

Despite the lack of evidence for symptomatic human infection with Maguari virus (MAGV), its close relation to Cache Valley virus (CVV), which does infect humans, remains a concern. We sequenced the complete genome of a MAGV-like isolate (OBS6657) obtained from a febrile patient in Pucallpa, Ucayali, Peru, in 1998. To facilitate its classification, we generated additional full-length sequences for the MAGV prototype strain, 3 additional MAGV-like isolates, and the closely related CVV (7 strains), Tlacotalpan (1 strain), Playas (3 strains), and Fort Sherman (1 strain) viruses. The OBS6657 isolate is similar to the MAGV prototype, whereas 2 of the other MAGV-like isolates are located on a distinct branch and most likely warrant classification as a separate virus species and 1 is, in fact, a misclassified CVV strain. Our findings provide clear evidence that MAGV can cause human disease.

Maguari virus (MAGV) was first isolated from a mixed pool of mosquitoes collected in Utinga forest in Brazil in 1957 and has since been isolated from a variety of mosquito species (including *Aedes* spp., *Anopheles* spp., *Culex* spp., *Wyeomyia* spp., and *Psorophora* spp.) in Ecuador, Brazil, Trinidad and Tobago, Colombia, Argentina, and French Guiana (*1*). MAGV also has been isolated from horses in Guyana and Colombia (*2*,*3*) and from sentinel mice in Brazil (*1*), with further serologic evidence for infection with MAGV-like viruses in cattle, water buffalo, sheep, and birds (*1*). However, there had been no clear evidence of symptomatic human infection with MAGV.

In 2015, an orthobunyavirus isolate from a patient with febrile illness that had been tentatively classified as MAGV on the basis of serologic reactivity by indirect immunofluorescence test was shown instead to be a new Caraparu (Group C) reassortant, for which the name Itaya virus has been proposed (*4*). However, serologic evidence of infection with MAGV-like viruses in humans has been reported in Argentina, Brazil, Peru, Colombia, and French Guiana (*1*,*5*,*6*). Furthermore, MAGV is considered a subtype of Cache Valley virus (CVV), which sometimes causes symptomatic human infection (*7*–*9*), reinforcing lingering concerns about the possible pathogenicity of MAGV for humans.

Given that cross-reactivity among bunyaviruses when using serologic techniques is not uncommon, their identification has increasingly involved incorporating genetic approaches to characterize these virus isolates. Such serologic cross-reactivity appears particularly to be an issue with MAGV, which was originally reported as CVV, and also shows cross-reactivity to other closely related viruses (*1*). Therefore, to clarify the genetic relationship between MAGV or MAGV-like viruses and the closely related CVV, as well as their association with acute human disease, we determined the complete genome sequences for all available MAGV isolates and conducted a comprehensive sequencing analysis of several full-length reference sequences for CVV strains and available strains of the closely related Tlacotalpan virus (TLAV), Playas virus (PLAV), and Fort Sherman virus (FSV) to facilitate the reliable classification of these and related virus isolates. 

## Materials and Methods

### Viruses

**Figure 1:**
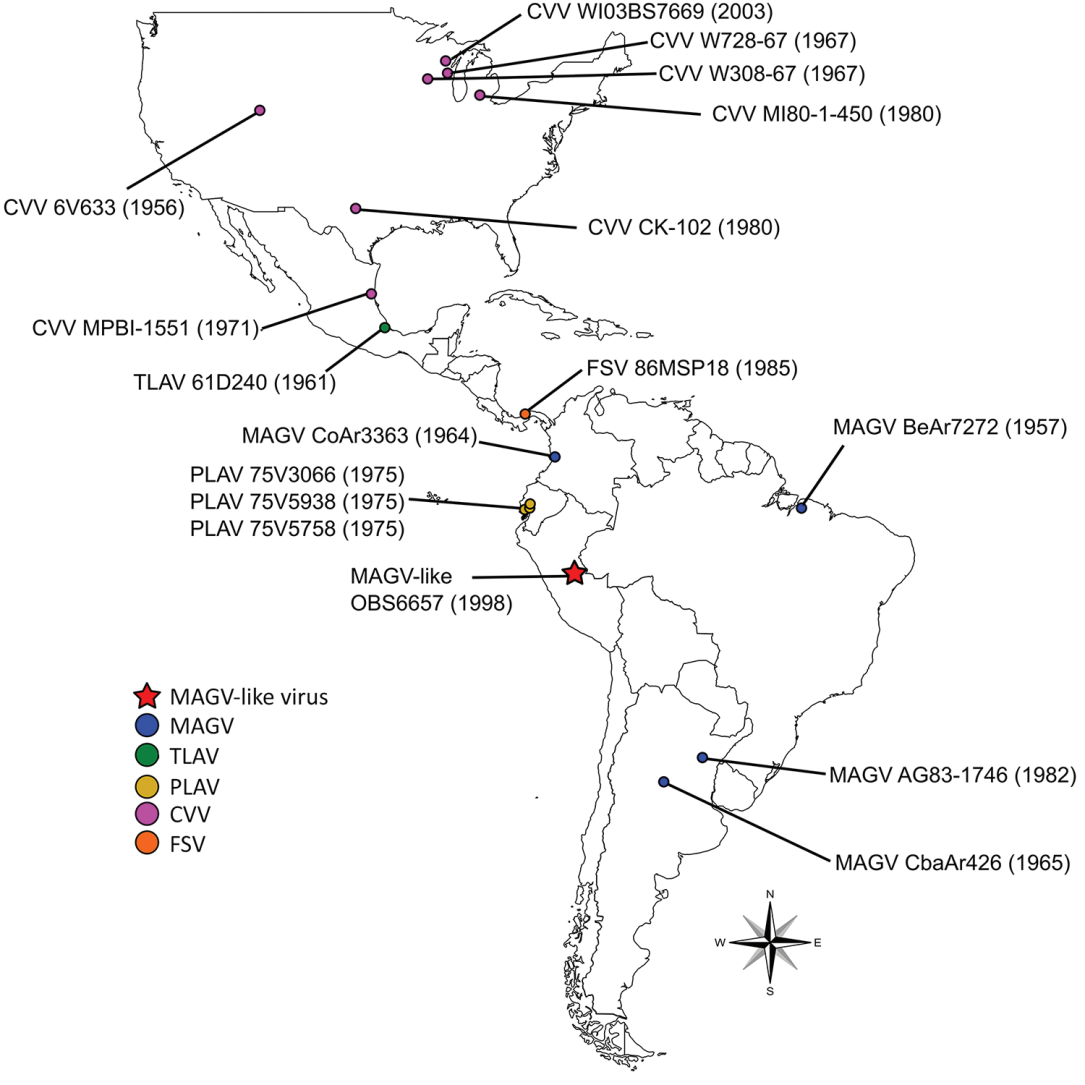
Geographic locations from which virus strains used in study of human infection with MAGV were isolated. Red star indicates the location of the MAGV-like isolate OBS6657; circles indicate source locations for other isolates used in this study. CVV, Cache Valley virus; FSV, Fort Sherman virus; MAGV, Maguari virus; PLAV, Playas virus; TLAV, Tlacotalpan virus.

We obtained 4 isolates identified as MAGV (including the prototype strain BeAr7272), spanning 1957–1998 and originating from 3 of the 7 countries from which the virus has been isolated (Colombia, Brazil, and Argentina) (Figure 1). Isolates were obtained from the World Reference Center for Emerging Viruses and Arboviruses (WRCEVA) or the Division of Vector-Borne Diseases, National Center for Emerging and Zoonotic Infectious Diseases, Centers for Disease Control and Prevention (DVBD-CDC, Atlanta, GA, USA) (Technical Appendix Table 1). In addition, we obtained 7 isolates of CVV spanning 1956–2003 and originating from diverse locations throughout the United States and Mexico, as well as 1 isolate of Tlacotalpan virus (Mexico), 3 isolates of Playas virus (Ecuador), and 1 isolate of FSV (Panama) (Figure 1) from either WRCEVA or DVBD-CDC. An additional MAGV-like virus strain, isolated from a patient exhibiting fever, headache, myalgia, and chills in Pucallpa, Ucayali, Peru, in 1998 (strain OBS6657), was obtained from WRCEVA. This isolate was collected under the terms of a human use protocol (NMRCD.2000.0006), which, along with the consent procedure, were approved by the Naval Medical Research Center Institutional Review Board in compliance with all US and Peruvian federal regulations governing the protection of human subjects.

### Sequencing

We extracted viral RNA using the QIAamp viral RNA mini kit (QIAGEN, Hilden, Germany) according to the manufacturer’s instructions with reverse transcription PCR (RT-PCR) reactions performed using the Superscript III reverse transcription kit (Life Technologies, Carlsbad, CA, USA) and the iProof High-Fidelity PCR Kit (Bio-Rad Laboratories, Hercules, CA, USA). We obtained preliminary virus genome sequences of the MAGV BeAr7272 and AG83–1746 strains, as well as PLAV (strain 75V3066), TLAV (strain 61D240), and FSV (strain 86MSP18), using the 454 FLX pyrosequencing technology platform (454 Life Sciences [Roche], Branford, CT, USA). We constructed libraries using previously described methods (*10*) and assembled and analyzed genomes using various publically available algorithms. Based on the preliminary 454-determined sequences, we designed primer sets to enable confirmation by Sanger sequencing, including analysis of additional strains of MAGV and PLAV. CVV strains were similarly sequenced by using primers based on complete sequences of the MNZ-92011 strain (GenBank accession nos. KC436108 [small (S) segment], KC436107 [medium (M) segment], KC436106 [large (L) segment]). The sequences of the noncoding regions, including the conserved genome termini, were amplified by using 3′ and 5′ rapid amplification of cDNA ends based on ligation-anchored PCR, as previously described (*11*–*13*). We deposited complete genome sequences determined in this study in GenBank (Technical Appendix Table 1); primer sequences are available on request.

### Phylogenetic and Sequence Analyses

Phylogenetic and sequence divergence analyses were conducted on the open reading frames (ORFs) and their corresponding amino acid sequences. These analyses incorporated the sequences for MAGV, CVV, TLAV, PLAV, and FSV determined in this study and additional representative orthobunyavirus sequences available in GenBank (Technical Appendix Table 2).

**Figure 2:**
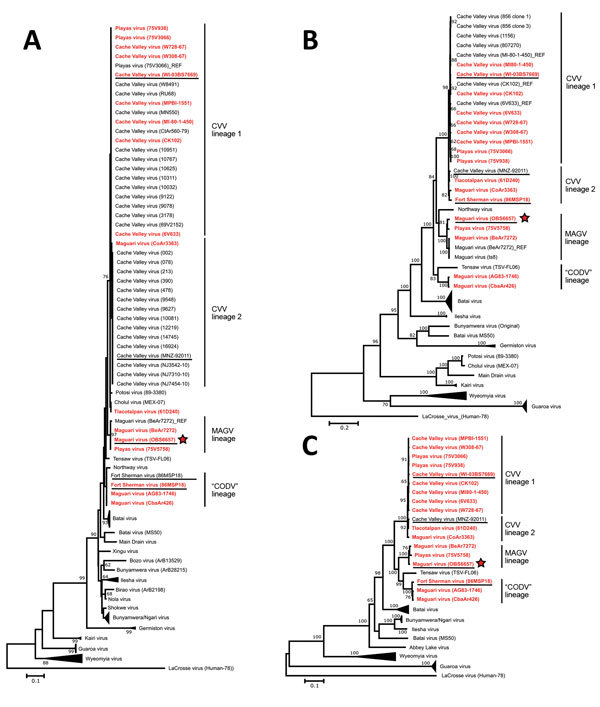
Phylogenetic relationship of MAGV-like isolate OBS6657 to other MAGV and CVV isolates and reference orthobunyaviruses. Maximum-likelihood trees (Jones, Taylor, and Thornton model, gamma-distributed) were constructed on the basis of the amino acid sequences of the nucleoprotein (A), glycoprotein (B), and polymerase (C). Bootstrap values based on 1,000 replicates are indicated for values >60. Sequences generated in this study are shown in red bold. Human isolates within the CVV, MAGV, and Córdoba virus clades are underlined, and the OBS6657 isolate is indicated with a red star. Scale bars indicate nucleotide substitutions per site. CVV, Cache Valley virus; CODV, Córdoba virus; MAGV, Maguari virus.

Sequences for each ORF or corresponding amino acid sequence were aligned using the MUSCLE algorithm, and the evolutionary history for each tree construction was inferred using the neighbor-joining (*14*) and maximum-likelihood (*15*) methods, as implemented in MEGA5 (*16*). For neighbor-joining analysis of amino acid sequences, we used a Poisson model (*17*) and uniform rates; for analysis of nucleotides, uniform rates also were specified. We computed evolutionary distances using the maximum composite likelihood method (*18*). For maximum-likelihood analysis of amino acids the Jones, Taylor, and Thornton model (JTT) (*19*), gamma-distributed (+ G) was used for all 3 segments. Alternatively, for both the amino acid and nucleotide analyses, we used the best fit model for each segment, based on the calculated Bayesian information criterion, as determined using the Model Selection Tool implemented in MEGA5 (*16*) (i.e., JTT + G [N amino acid]; Le-Gascuel [LG] model [*20*] + G plus invariant sites [+ I] [LG + G + I; GPC amino acid], LG + G, + I plus frequencies [LG + G + I + F; L amino acid] or Tamura 3-parameter [*21*] + G [T92 + G, N nucleotide]; general time reversible [*22*] + G + I [GTR + G + I; GPC and L nucleotide]). Evaluation of statistical support for the neighbor-joining and maximum-likelihood tree topologies was based on bootstrap resampling (*23*); values were calculated on the basis of 1,000 replicates, and values >60 are indicated. We conducted an additional analysis based on Bayesian inference using Mr Bayes version 3 (*24*,*25*), as implemented in TOPALi version 2 (*26*) with the best fit model for each segment selected on the basis of the calculated Bayesian information criterion, using the integrated model selection tool in TOPALi (i.e., JTT + G [N amino acid]; JTT + G + I [GPC and L amino acid] or GTR + G [N amino acid]; GTR + G + I [GPC and L amino acid]). Posterior probability values >0.60 were indicated. The use of these different methods and models did not produce substantially different tree topologies with respect to the virus lineages under study (Figure 2; Technical Appendix Figures 1, 2).

We calculated sequence divergence values on the basis of nucleotide and amino acid alignments constructed with ClustalW as implemented in MegAlign (LaserGene 12; DNASTAR, Madison, WI, USA). Furthermore, an additional recombination analysis was performed on concatenated (S, M, and L) genome sequences of all CVV, MAGV, PLAV, TLAV, and FSV isolates where full-length sequences were available. Initial alignment was performed by using the MUSCLE algorithm, as implemented in MEGA5 (*16*), and these data were then further analyzed by using the RDP (*27*), GENECONV (*28*), BOOTSCAN/RECSCAN (*29*), MAXCHI (*30*), CHIMAERA (*31*), SISCAN (*32*), and 3SEQ (*33*) programs, as implemented in RDP4 Beta 4.83 (*34*).

## Results and Discussion

### Analysis of MAGV and MAGV-Like Sequences

In our phylogenetic analysis, the MAGV isolates we examined form 2 distinct clades: 1 contains the prototype strain BeAr7272 and “PLAV” (strain 75V5758), the other comprises the AG83-1746 strain and the CbaAr426 strain (Figure 2). Both of these atypical “MAGV” isolates (AG83-1746 and CbaAr426) were collected in Argentina and were isolated 17 years apart, indicating that this virus most likely continues to stably circulate in that region. MAGV (CoAr3363) was found to be an incorrectly classified isolate of CVV and thus is not considered further in this section. Of particular interest to the aims of this study was the MAGV-like isolate designated OBS6657, which was previously identified by complement fixation as a MAGV-like virus, after isolation from a symptomatic patient in Peru. Phylogenetic analysis indicates that this virus is closely related to the prototype MAGV isolate BeAr7272, with which it shares a clade.

Our divergence analysis comparing the 2 groups formed by the isolates originally identified as MAGV showed 95.7% (N), 84.3%–85.2% (GPC), and 90.1%–90.7% (L) amino acid divergence. The extremely low level of N protein variability seems to be a characteristic of these and related virus groups, and the L protein divergence values are barely above the 10%-aa divergence sometimes suggested as a cutoff for speciation. However, the glycoprotein, which is the most variable of the viral protein products, differs substantially from all other related virus groups and also between these 2 groups. Furthermore, the consistent evidence for separate evolutionary histories inferred by the phylogenies for all 3 proteins indicates that these viruses most likely differ sufficiently to warrant reclassification of the AG83-1746 and CbaAr426 strains as a distinct virus species. In accordance with the prevailing naming conventions for bunyaviruses, which dictate naming based on the location of initial isolation, we use “Córdoba virus” (CODV) and “CODV lineage” herein to refer to viruses belonging to this group and to distinguish them from the canonical MAGV strains.

In contrast to these various misidentified viruses, the OBS6657 isolate clearly grouped with the prototype MAGV strain BeAr7272 and showed 100% (N), 97.1% (GPC), and 99.0% (L) amino acid identity, respectively, across each of the three major gene products, despite being isolated >40 years later. Given that this virus was isolated from a patient exhibiting clear evidence of an acute febrile infection, our data provide strong evidence that MAGV infection can cause clinical disease in humans. In light of this evidence, and particularly when considered with serologic data that indicate frequent infection with MAGV-like viruses (2%–64% positive serum samples) (*1*,*5*,*6*) in South America, it appears likely that the failure to recognize MAGV as a causative agent of febrile infection in humans is due to a lack of systematic surveillance and diagnostic testing rather than a lack of pathogenic potential. Furthermore, the inclusion of PLAV (75V5758) in this group suggests that, even if human virulence were restricted to this group, the affected region might cover a large portion of Central and South America, including Brazil (strain BeAr7272), Peru (strain OBS6657), and Ecuador (PLAV strain 75V5758).

### Analysis of CVV Sequences

To obtain sufficient data to provide a reliable framework for analyzing the MAGV isolates in this study, and in light of their recently demonstrated potential as human pathogens, we further conducted full-genome sequence analysis for several CVV strains and for the closely related PLAV and TLAV, and for the human pathogenic FSV, which also has been described to be closely related to CVV (*35*). Of the 7 CVV isolates that we fully sequenced, all appear to belong to the recently described lineage 1 of CVV, consistent with the suggestions that older strains from the United States, such as those used in this study, belong to this lineage and have only recently been supplanted in some areas by lineage 2 viruses (*36*). In addition, TLAV (strain 61D240) and 2 of the 3 strains of PLAV virus analyzed (strains 75V3066 and 75V5938) clearly grouped within the CVV clade for all 3 segments (Figure 2). The other PLAV strain (strain 75V5758) belonged to the canonical MAGV lineage, as noted earlier. The need to reclassify these virus strains as isolates of CVV is further supported by divergence analysis (Technical Appendix Figure 3, panels A–C), which shows that each of these viruses shows >90% aa identity to all known CVV isolates for all 3 major proteins, including the highly variable glycoprotein. Given the surprising nature of this result, we further confirmed this finding using multiple virus passages and stocks provided from 2 different collections (WRCEVA and DVBD-CDC); however, the results remained consistent (data not shown). Overall, our data provide unequivocal support, on the basis of full-genome sequencing data for all 3 segments of multiple virus strains, for previous observations from S-segment data from single strains of each of TLAV and PLAV (*36*) that had suggested these strains might indeed have been misclassified as new virus species upon discovery. Similarly, we also identified 1 isolate previously classified as MAGV (strain CoAr3363) as a misidentified CVV lineage 2 virus (Figure 2; Technical Appendix Figure 3, panels A–C).

We also included FSV in our analysis. This isolate was collected in 1985 at Fort Sherman, a former US Army base in Panama, from a patient exhibiting fever (101°F), malaise, muscle aches, and sore throat. The isolate cross-reacted very closely with CVV, MAGV, and PLAV virus in complement fixation but only with CVV and PLAV by plaque-reduction neutralization test (*37*). During the original isolation, FSV was suggested to be a subtype of CVV (*35*). However, sequencing of the S segment has shown it to be only moderately related to CVV, although statistical support for the branching arrangement was poor (*38*). On the basis of our full-length analysis of all 3 gene segments, we can now clarify and reconcile these apparently disparate findings, by identifying FSV as a reassortant between CVV lineage 2 and “CODV” (CODV_S_/CVV_M_/CODV_L_). Taken together, the phylogenetic arrangement (Figure 2) and divergence analysis (Technical Appendix Figure 3, panels A–C), as well as a recombination/reassortment analysis using concatenated genomes (Technical Appendix Figure 3, panel D), indicate that CODV (CbaAr426) is the closest relative of the S and L segment donor, and the strain CoAr3363 (previously misclassified as MAGV) from CVV lineage 2 is the closest to the M segment donor. This observation would appear to explain the close antigenic relationship of FSV to both CVV and MAGV by complement fixation but not plaque-reduction neutralization test, as well as the lack of sequence relationship between the S segments of these viruses.

One important consequence of the genetic reassignment of not only TLAV and PLAV, but also of MAGV (CoAr3363) to the CVV group, and identification of FSV as a previously unrecognized CVV reassortant, is to substantially expand the known geographic range of CVV. On the basis of these data, the range of CVV must now be considered to include not only the United States and northern and central Mexico but probably all of Central America and the northern areas of South America. Furthermore, although both PLAV strains appear to belong to CVV lineage 1, TLAV belongs to CVV lineage 2, as does the CVV donor of the FSV M segment. This finding suggests that both lineages exist in Central America and that lineage 2 has been circulating in that region since at least the 1960s, when the first of these viruses were isolated. This supposition is also supported by the assignment of the MAGV CoAr3363 strain, which was isolated in 1964, to lineage 2. Taken together, these findings suggest that although these CVV lineage 2 strains have only recently emerged in the eastern United States, they might in fact have been circulating in South America for decades and have only recently been introduced into the United States from these regions.

### Technical Considerations in the Genetic and Serologic Analysis of Nucleoprotein Sequences

Our analysis of the ORF and amino acid data identified several distinct clades representing CVV (lineages 1 and 2) and 2 separate clades of what had been identified as MAGV strains (Figure 2). These clades were all well-supported for the GPC and L ORFs. However, although the members of the identified groups also were suggested to remain consistent when data for the nucleoprotein were analyzed, suggesting a lack of reassortment in these viruses, the branching was only poorly supported. This observation most likely resulted from an overall low level of total variation between these closely related groups, which is exacerbated when shorter datasets, such as that for N, are used. It is possible (and maybe even likely) that this issue will continue to present a growing problem for orthobunyavirus group analyses based on N as an increasing number of sequences for related virus groups continue to become available.

The extent of this issue is further highlighted by our divergence analysis (Technical Appendix Figure 3, panels A–C), which shows extremely high sequence identity (93.6%–100% aa) among members of these groups, even when the analysis includes more distantly related and clearly geographically distinct viruses, such as Batai virus. These high levels of conservation may then also suggest a basis for the historical difficulties in serologic virus identification that seem to be increasingly identified during retrospective virus identification efforts, including this one. Surprisingly, a recent report showed that a virus isolate originally reported as MAGV was, in fact, a reassortant of Caraparu, a group C virus (*4*), which is genetically only distantly related to the Bunyamwera serogroup viruses. This finding suggests that the accurate serologic identification of orthobunyaviruses is difficult. This observed lack of overall variability in the N proteins of these viruses seems to suggest that the serologic identification of Bunyamwera group viruses, and perhaps also other groups, by complement fixation might be particularly difficult, if not impossible, and needs to be approached carefully. Furthermore, given the apparent extent of this problem and the widespread availability and modest costs associated with RT-PCR and Sanger sequencing, it may be prudent to recommend routinely incorporating at least partial genetic characterization as a part of the identification process for all new bunyavirus isolates. Indeed, given the high level of conservation observed within the N gene of related orthobunyavirus groups, it would make an ideal target for the development of broadly cross-reactive RT-PCR primer sets for diagnostic applications.

In summary, we identified MAGV as the causative agent of a human febrile infection in Peru and showed that the virus associated with this infection is highly similar to the prototype MAGV isolate, suggesting that other viruses of this lineage also might have pathogenic potential in humans. In addition, our in-depth analysis of the closely related CVV group and other similar viruses showed that PLAV and TLAV do not exist as distinct virus species but are misclassified strains of other existing groups and that the human pathogenic FSV is a previously unrecognized reassortant between CVV, itself a known human pathogen, and a new lineage whose members had previously been identified as MAGV but are genetically distinct from the lineage occupied by the prototypical MAGV isolates. On the basis of its location of origin, we suggest the name “Córdoba virus” for this new lineage. Based on the reclassification of these virus isolates, it is clear that the endemic region for CVV is much larger than previously recognized and that CVV and MAGV most likely are responsible for unrecognized febrile infections throughout North Central, and South America. We hope that a better understanding of the genetic relationships between bunyavirus groups, particularly in relation to their relevance for human infection, coupled with an increased availability of reference genome sequence information, such as provided by this study, will help enable future surveillance and diagnostic efforts and increase awareness of the importance of orthobunyaviruses as human pathogens.

Technical Appendix Source and sequence information for Maguari virus strains sequenced in this study; GenBank accession numbers for reference orthobunyavirus sequences used in the phylogenetic analyses; comparison of phylogenetic analyses of nucleoprotein, glycoprotein and polymerase amino acid sequences; comparison of phylogenetic analyses of nucleoprotein, glycoprotein and polymerase open reading frame nucleotide sequences; divergence analyses for nucleoprotein, glycoprotein, and polymerase sequences.
