# Supplementary material for: Maguari Virus Associated with Human Disease
Source: Emerg Infect Dis. 2017 Aug;23(8):1325–31. doi: 10.3201/eid2308.161254 (PMC5547800; doi:10.3201/eid2308.161254)
Supplement: Technical Appendix — Source and sequence information for Maguari virus strains sequenced in this study; GenBank accession numbers for reference orthobunyavirus sequences used in the phylogenetic analyses; comparison of phylogenetic analyses of nucleoprotein, glycoprotein and polymerase amino acid sequences; comparison of phylogenetic analyses of nucleoprotein, glycoprotein and polymerase open reading frame nucleotide sequences; divergence analyses for nucleoprotein, glycoprotein, and polymerase sequences. [file 16-1254-Techapp-s1.pdf]

Article DOI: https://dx.doi.org/10.3201/eid2308.161254

Maguari Virus Associated with Human Disease

Technical Appendix

Technical Appendix Table 1. Source and sequence information for virus strains sequenced in this study

Virus/strain	Host	Year	Location	GenBank accession no.
Maguari virus				
BeAr 7272 prototype	Mixed mosquito pool*	1957	Utinga forest, Pará, Brazil	S: KX100103<br>M: KX100104<br>L: KX100105
CoAr 3363	*Aedes scapularis*	1964	Buenaventura, Valle del Cauca, Colombia	S: KX100106<br>M: KX100107<br>L: KX100108
CbaAr 426	*Ae. albifasciatus*	1965	Córdoba, Argentina	S: KX100109<br>M: KX100110<br>L: KX100111
AG83–1746	*Psorophora varinervis*	1982	*Calchaquí* Forest, Santa Fe, Argentina	S: KX100112<br>M: KX100113<br>L: KX100114
Maguari-like virus: OBS 6657	Human	1998	Pucallpa, Ucayali, Peru	S: KX100115<br>M: KX100116<br>L: KX100117
Tlacotalpan virus: 61D240	*Mansonia titillans*	1961	Tlacotalpan, Veracruz, Mexico	S: KX100118<br>M: KX100119<br>L: KX100120
Playas virus				
75V3066	*Ae. taeniorhynchus*	1975	Playas, Ecuador	S: KX100121<br>M: KX100122<br>L: KX100123
75V5938	*Aedeomyia (ochler) taeniorhynchus*	1975	Guayaquil, Ecuador	S: KX100124<br>M: KX100125<br>L: KX100126
75V5758	*Aedeomyia (ochler) taeniorhynchus*	1975	La Florida, Ecuador	S: KX100127<br>M: KX100128<br>L: KX100129
Fort Sherman virus: 86MSP18	Human	1985	Fort Sherman, Panama	S: KX100130<br>M: KX100131<br>L: KX100132
Cache Valley virus				
6V633 prototype	*Culiseta inornata*	1956	Cache Valley (near Wellsville), Utah, USA	S: KX100133<br>M: KX100134<br>L: KX100135
W728–67	*Ae. communis*	1967	Mazomanie, Wisconsin, USA	S: KX100136<br>M: KX100137<br>L: KX100138
W308–67	*Ae. trivittatus*	1967	Wyalusing, Wisconsin, USA	S: KX100139<br>M: KX100140<br>L: KX100141
MPB1–1551	*Psorophora confinnis*	1971	Palo Blanco, Tamaulipas, Mexico	S: KX100142<br>M: KX100143<br>L: KX100144
CK-102	Sheep (sentinel)	1980	San Angelo, Texas, USA	S: KX100145<br>M: KX100146<br>L: KX100147
MI80–1-450	Horse	1980	Cass County, Michigan, USA	S: KX100148<br>M: KX100149<br>L: KX100150
WI-03BS7669	Human	2003	Wisconsin, USA	S: KX100151

Virus/strain	Host	Year	Location	GenBank accession no.
				M: KX100152<br>L: KX100153

*Contained *Aedes scapularis*, *Ae serratus*, *Ae sexlineatus*, *Mansonia* spp. and *Psorophora ferox*.

Technical Appendix Table 2. GenBank accession numbers for reference orthobunyavirus sequences used in the phylogenetic analyses

Group/virus	Strain	GenBank accession nos.		
		S segment	M segment	L segment
Bunyamwera				
Abbey Lake virus	Cu20-XJ	KJ710424	KJ710423	KJ710425
Batai virus	MM2222	JX846595	JX846596	JX846597
	8627–11	FJ436802	FJ436799	
	804922	FJ436805		
	804986	FJ436803	FJ436798	
	804988	FJ436800	FJ436804	
	IG 20217 (Chittoor)	JX846598	JX846599	JX846600
	Italy-2009	KC168046	KC168047	KC168048
	NM-12	KJ187040	KJ187039	KJ187038
	ON-1-E-94	AB257761	AB257764	
	ON-7-B-01	AB257762	AB257765	
	UgMP6830	JX846601	JX846602	JX846603
	XQ-B	KJ398936		
	MS50	JX846604	JX846605	JX846606
	Calovo 134	KJ542624	KJ542625	KJ542626
	Calovo 184		DQ334335	
	Calovo 8020	KJ542630	KJ542631	KJ542632
	Calovo 8040	KJ542633	KJ542634	KJ542635
	Calovo JAn MS3	KJ542627	KJ542628	KJ542629
Birao virus	ArB2198	AM711131		
Bozo virus	ArB13529	AM711132		
Bunyamwera virus	Original	NC_001927	NC_001926	NC_001925
	ArB28215	AM711130		
	ArB29051	AM709778		
Cache Valley virus	MNZ-92011	KC436108	KC436107	KC436106
	002	GU018033		
	6V633		AF082576	
	69V2152	KP835919		
	078	GU018034		
	213	GU018035		
	390	GU018036		
	478	GU018037		
	3178	KP835920		
	9078	KP835921		
	9122	KP835922		
	9548	KP835930		
	9627	KP835923		
	10032	KP835931		
	10081	KP835932		
	10311	KP835933		
	10625	KP835924		
	10767	KP835934		
	10951	KP835935		
	12219	KP835936		
	14745	KP835928		
	16924	KP835929		
	CK-102		AF186242	
	CtAr560–79	KP835925		
	MI80–1-450		AF186241	
	MN550	KP835926		
	NJ3542–10	KF296339		
	NJ7310–10	KF296340		
	NJ7454–10	KF296341		
	RU68	KP835927		
	W8491	KP835937		
Cholul virus	MEX-07	EU879062	JN808310	
Fort Sherman virus	86MSP18	EU564829		

Group/virus	Strain	GenBank accession nos. S segment	M segment	L segment
Germiston virus	SAAr1050	M19420	M21951	
Ilesha virus	8e	KC608151	KC608150	KC608149
	R5964	AY729651	KF234074	KF234075
	KO/2		AY859372	
	ArB16282	AM709780		
	HB80P125	AM709779		
Kairi virus	MEX-07	EU879063	GQ118699	
	TRVL8900	X73467	EU004186	
Maguari virus	BeAr7272	D13783	AY286443	
Main Drain virus	BFS5015	X73469	EU004187	
Mboke virus	DakArY357	AY593727		
M'Poko virus	ArB365	AM711133		
Nola virus	ArB2882	AM711134		
Ngari virus	9800521	JX857325	JX857326	JX857327
	9800535	JX857328	JX857329	JX857330
	Adrar	KJ716848	KJ716849	KJ716850
	DaKArD28542	JX857316	JX857317	JX857318
	GSA-TS7–5170	KM507341	KM514677	KM507336
	SUD HKV66	JX857319	JX857320	JX857321
	SUD HKV141	JX857322	JX857323	JX857324
	ISL-TS2–5242	KM507342	KM514678	KM507334
	TND-S1–19801	KM507343	KM514679	KM507335
Northway virus	0234	X73470	EU004188	
Playas virus	75V3066	KP83593		
Potosi virus	89–3380	AY729652	EU004189	
Shokwe virus	SAAr4042	EU564831		
Tensaw virus	TSV-FL06	FJ943507	FJ943506	FJ943509
Wyeomyia/Anhembi				
Anhembi virus	SPAr2984	JN572064	JN572063	JN572062
Cachoeira Porteira virus	BeAr328208	JN968592	JN968591	JN968590
Iaco virus	BeAr314206	JN572067	JN572066	JN572065
Macaua virus	BeAr306329	JN572070	JN572069	JN572068
Sororoca virus	BeAr32149	JN572073	JN572072	JN572071
Taiassui virus	BeAr671	JN572076	JN572075	JN572074
Tucunduba virus	BeAr278	JN572079	JN572078	JN572077
Wyeomyia virus	Original	JN572082	JN572081	JN572080
	TRVL8349	JN801033	JN801034	JN801035
	Darien	JN801036	JN801037	JN801038
Guaroa virus				
	ASA1165	KM245552	KM245553	KM245554
	BeH22063	KM245522	KM245523	KM245524
	CoH352111	KM245519	KM245520	KM245521
	FPI01900	KM245546	KM245547	KM245548
	FSJ2035	KM245549	KM245550	KM245551
	FVB0546	KM245528	KM245529	KM245530
	FVB0840	KM245537	KM245538	KM245539
	FVB0849	KM245540	KM245541	KM245542
	FVB2032	KM245543	KM245544	KM245545
	IQD8537	KM245525	KM245526	KM245527
	MIS0239	KM245534	KM245535	KM245536
	OBT5637	KM245531	KM245532	KM245533
California encephalitis (outgroup): LaCrosse virus	Human-78	NC_004110	NC_004109	NC_004108

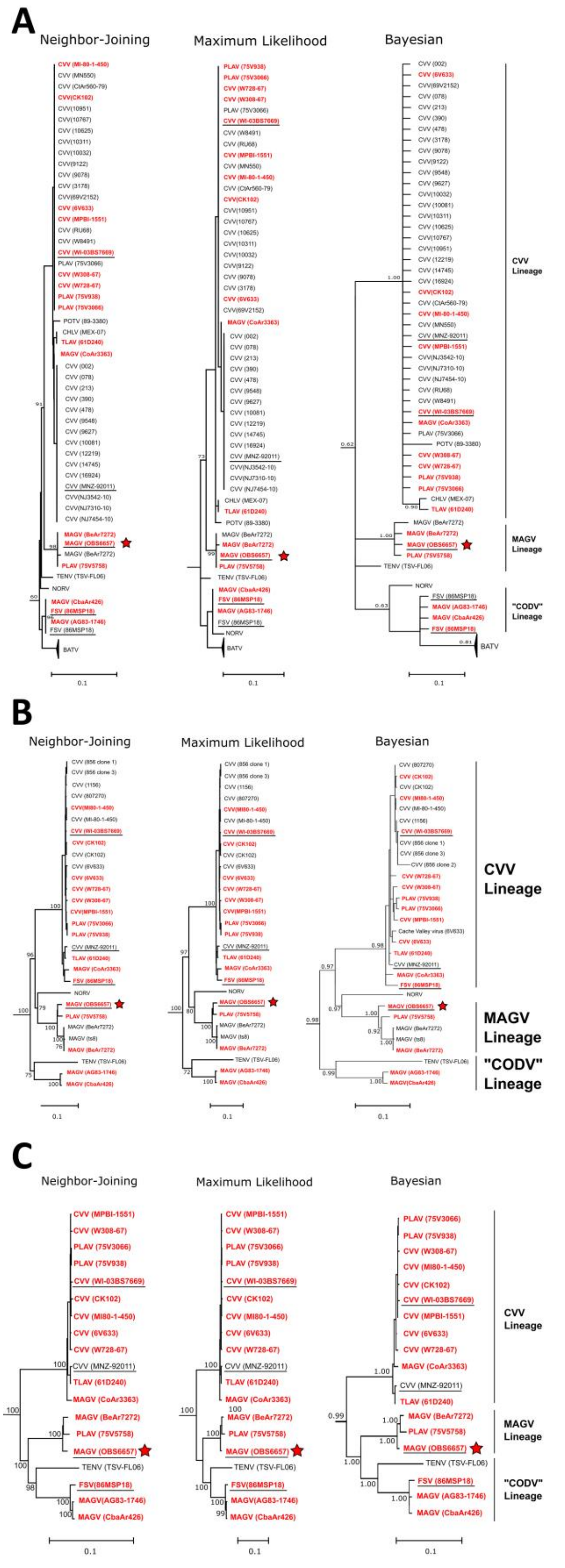
A
Neighbor-Joining
Maximum Likelihood
Bayesian
CVV Lineage
MAGV Lineage
"CODV" Lineage
B
Neighbor-Joining
Maximum Likelihood
Bayesian
CVV Lineage
MAGV Lineage
"CODV" Lineage
C
Neighbor-Joining
Maximum Likelihood
Bayesian
CVV (MPBI-1551)
CVV (W308-67)
PLAV (75V3066)
PLAV (75V938)
CVV (WI-03BS7669)
CVV (CK102)
CVV (MI80-1-450)
CVV (6V633)
CVV (W728-67)
CVV (MNZ-92011)
TLAV (61D240)
MAGV (CoAr3363)
MAGV (BeAr7272)
PLAV (75V5758)
MAGV (OBS6657)
TENV (TSV-FL06)
FSV(86MSP18)
MAGV(AG83-1746)
MAGV (CbaAr426)
CVV Lineage
MAGV Lineage
"CODV" Lineage
0.1

Technical Appendix Figure 1. Comparison of phylogenetic analyses of (A) nucleoprotein, (B) glycoprotein, and (C) polymerase amino acid sequences generated using various phylogenetic methods and models. Neighbor-joining trees for all 3 segments were generated using the Poisson model with uniform rates. Maximum-likelihood trees were constructed based on the best fitting model for each dataset (i.e., N = JTT+G; GPC = LG+G+I; L = LG+G+I+F). Bootstrap values for both methods were based on 1,000 replicates and are indicated if >60. Analysis by Bayesian inference also used the best fitting model for each dataset (i.e., N = JTT+G; GPC and L = JTT+I+G) and shows posterior probability values >0.60. For clarity, only the portion of the tree containing the sequences generated in this study (i.e., CVV, MAGV, and CODV lineages) are shown, and measures of statistical support for branching within the CVV lineage are omitted. Sequences generated in this study are shown in red and bold. The OBS6657 isolate is indicated with a red star and human-derived isolates are underlined. BATV, Batai virus; CVV, Cache Valley virus; CHLV, Cholul virus; CODV, Córdoba virus; FSV, Fort Sherman virus; MAGV, Maguari virus; MDV, Main Drain virus; NORV, Northway virus; PLAV, Playas virus; POTV, Potosi virus; TLAV, Tlacotalpan virus; TENV, Tensaw virus.

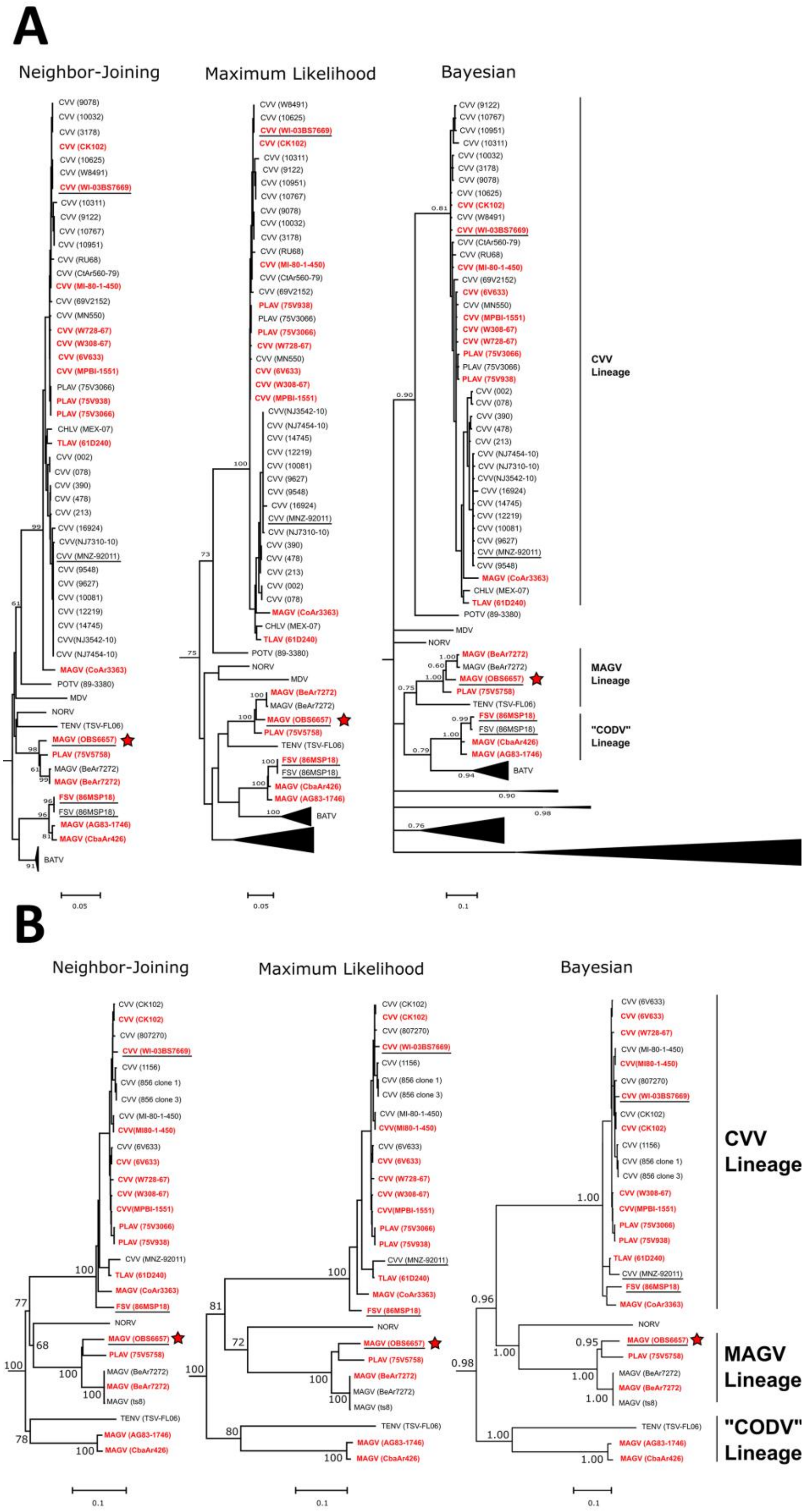
A
Neighbor-Joining
Maximum Likelihood
Bayesian
CVV Lineage
MAGV Lineage
"CODV" Lineage
CVV (9078)
CVV (10032)
CVV (3178)
CVV (CK102)
CVV (10625)
CVV (W8491)
CVV (WI-03BS7669)
CVV (10311)
CVV (9122)
CVV (10767)
CVV (10951)
CVV (RU68)
CVV (CtAr560-79)
CVV (MI-80-1-450)
CVV (69V2152)
CVV (MN550)
CVV (W728-67)
CVV (W308-67)
CVV (6V633)
CVV (MPBI-1551)
PLAV (75V3066)
PLAV (75V938)
CHLV (MEX-07)
TLAV (61D240)
CVV (002)
CVV (078)
CVV (390)
CVV (478)
CVV (213)
CVV (16924)
CVV(NJ7310-10)
CVV (MNZ-92011)
CVV (9548)
CVV (9627)
CVV (10081)
CVV (12219)
CVV (14745)
CVV(NJ3542-10)
CVV (NJ7454-10)
MAGV (CoAr3363)
POTV (89-3380)
MDV
NORV
TENV (TSV-FL06)
MAGV (OBS6657)
PLAV (75V5758)
MAGV (BeAr7272)
FSV (86MSP18)
MAGV (AG83-1746)
MAGV (CbaAr426)
BATV
0.05
0.1
B
CVV (807270)
CVV (1156)
CVV (856 clone 1)
CVV (856 clone 3)
CVV(MI80-1-450)
CVV(MPBI-1551)
MAGV (ts8)

Technical Appendix Figure 2. Comparison of phylogenetic analyses of nucleoprotein (A), glycoprotein (B), and polymerase (C) open reading frame nucleotide sequences generated using various phylogenetic methods and models. Neighbor-joining trees for all 3 segments were generated using uniform rates. Maximum-likelihood trees were constructed based on the best fitting model for each dataset (i.e. N = T92+G; GPC and L= GTR+G+I). Bootstrap values for both of these methods were based on 1,000 replicates and are indicated if >60. Analysis by Bayesian inference also used the best fitting model for each dataset (i.e., N = GTR+G; GPC and L = GTR+G+I) and shows posterior probability values >0.60. For clarity, only the portion of the tree containing the sequences generated in this study (i.e., CVV, MAGV, and CODV lineages) are shown, and measures of statistical support for branching within the CVV lineage are omitted. Sequences generated in this study are shown in red and are bold. The OBS6657 isolate is indicated with a red star, and human-derived isolates are underlined. BATV, Batai virus; CVV, Cache Valley virus; CHLV, Cholul virus; CODV, Córdoba virus; FSV, Fort Sherman virus; MAGV, Maguari virus; MDV, Main Drain virus; NORV, Northway virus, PLAV, Playas virus; POTV, Potosi virus; TLAV, Tlacotalpan virus; TENV, Tensaw virus.

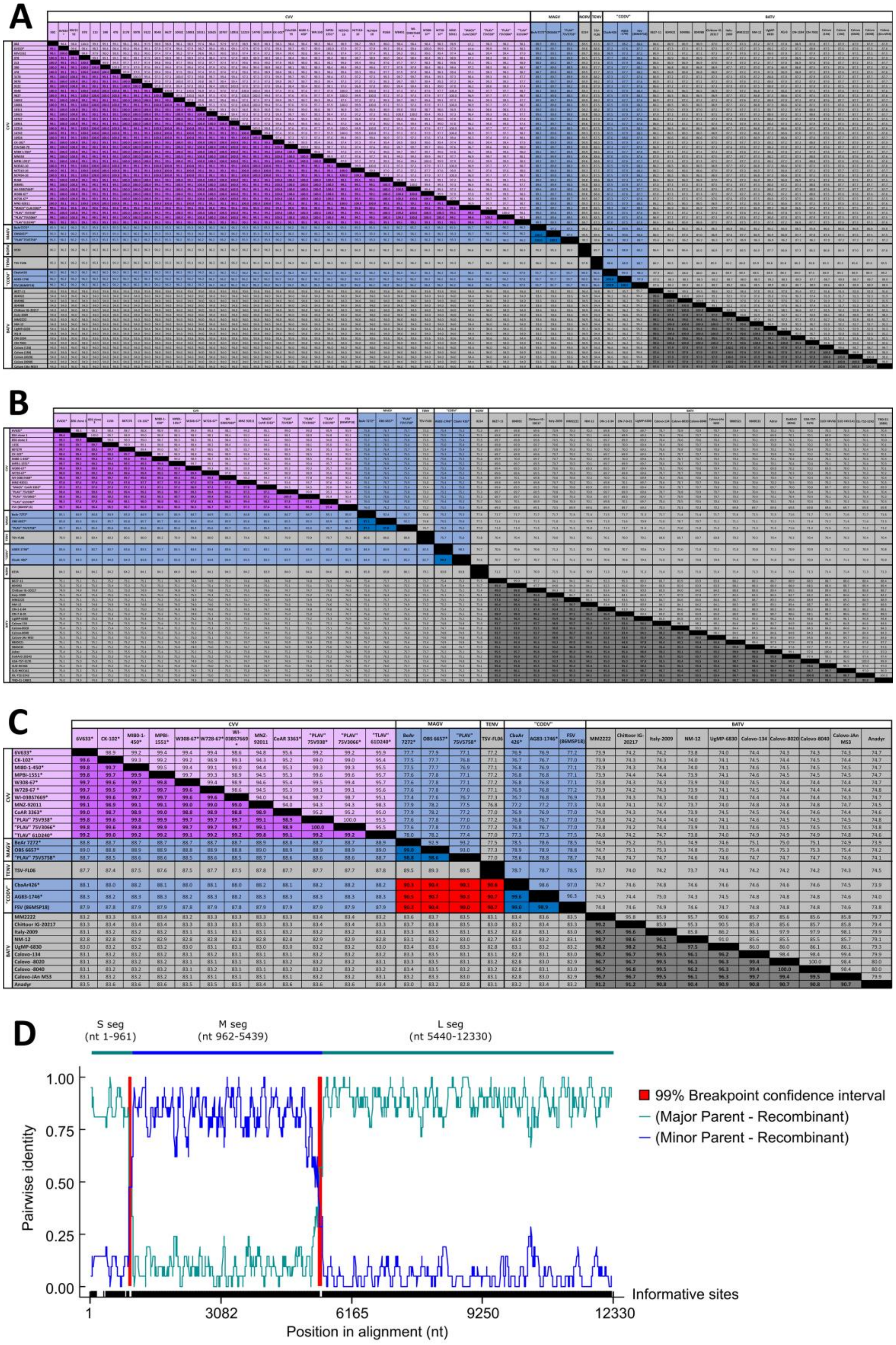
A
B
C
D
S seg
(nt 1-961)
M seg
(nt 962-5439)
L seg
(nt 5440-12330)
99% Breakpoint confidence interval
(Major Parent - Recombinant)
(Minor Parent - Recombinant)
Pairwise identity
Informative sites
Position in alignment (nt)
1
3082
6165
9250
12330
1.00
0.75
0.50
0.25
0.00

Technical Appendix Figure 3. Divergence analyses for nucleoprotein (A), glycoprotein (B), and polymerase (C) sequences. Nucleotide (top) and amino acid (bottom) sequences of MAGVs (blue) and CVVs (purple) and for other closely related reference sequences (gray). Amino acid identities among members of the same group are highlighted in dark shades of the corresponding colors. Although in the analysis of the nucleoprotein sequence all pairings showed identity values >90%, in the polymerase sequence analysis pairing outside of a clade that showed identity values >90% are highlighted in red. D) Recombination analysis. Concatenated full-length genomes for all members of the CVV, MAGV, and CODV lineages with available data were generated and analyzed for evidence of recombination using RDP4 Beta 4.83 (*1*). A single recombination event (equivalent to reassortment in the segmented virus) was identified and corresponded to the M segment junctions in the concatemer. The major parent (i.e., S and L segment donor) was identified as being most closely related to CODV strain CbaAr426 (shown in green), and the minor parent (i.e., M segment donor) was most closely related to CVV strain CoAr3363 (shown in blue). BATV, Batai virus; CVV, Cache Valley virus; CODV, Córdoba virus; FSV, Fort Sherman virus; MAGV, Maguari virus; NORV, Northway virus; PLAV, Playas virus; TLAV, Tlacotalpan virus; TENV, Tensaw virus.

Reference

1. Martin DP, Murrell B, Golden M, Khoosal A, Muhire B. RDP4: Detection and analysis of recombination patterns in virus genomes. Virus Evol. 2015;1:vev003. **PMID: 27774277**
